# Supplementary material for: Comparison of the Effect of Native 1,4-Naphthoquinones Plumbagin, Menadione, and Lawsone on Viability, Redox Status, and Mitochondrial Functions of C6 Glioblastoma Cells
Source: Nutrients. 2019 Jun 7;11(6):1294. doi: 10.3390/nu11061294 (PMC6628372; doi:10.3390/nu11061294)
Supplement: Supplementary file 1 [file nutrients-11-01294-s001.pdf]

SUPPLEMENTARY DATA

Comparison of the effect of native 1,4-naphthoquinons plumbagin, menadione and lawsone on viability, redox status and mitochondrial functions of C6 glioblastoma cells

Daiva Majiene [*], Jolita Kuseliauskyte, Arturas Stimbirys, Aiste Jekabsone

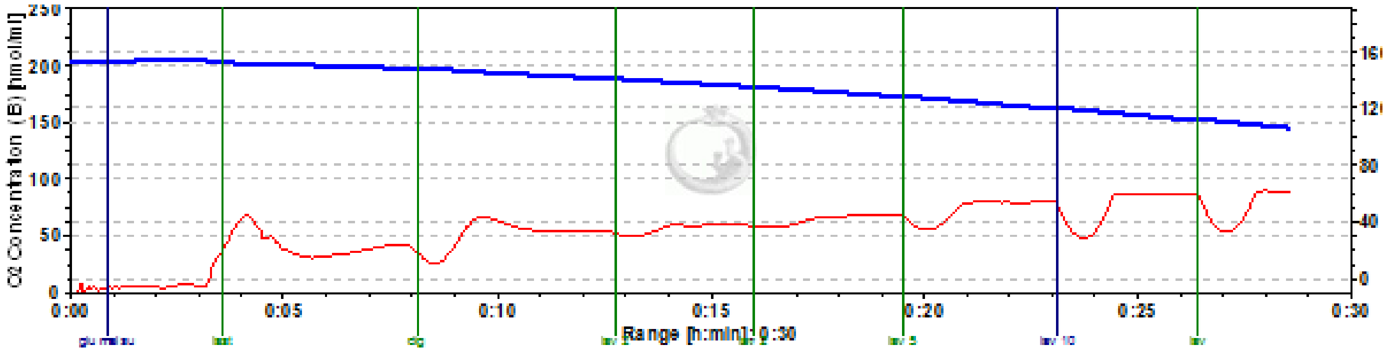

Figure S 1A. Typical traces of measurement of mitochondrial non-phosphorylating respiration rate in C6 cell.

At the beginning respiration rate with 2×10^{6} cells/chamber and in the presence of glutamate + malate (5 mM each) and succinate (12 mM) was recorded. Further additions: 10 µM of digitonin, 10 µM lawsone , 20 µM lawsone, 50 µM lawsone, 100 µM lawsone. The upper trace (blue) indicates oxygen concentration in a respiratory chamber, the lower (red) one – oxygen consumption rate.

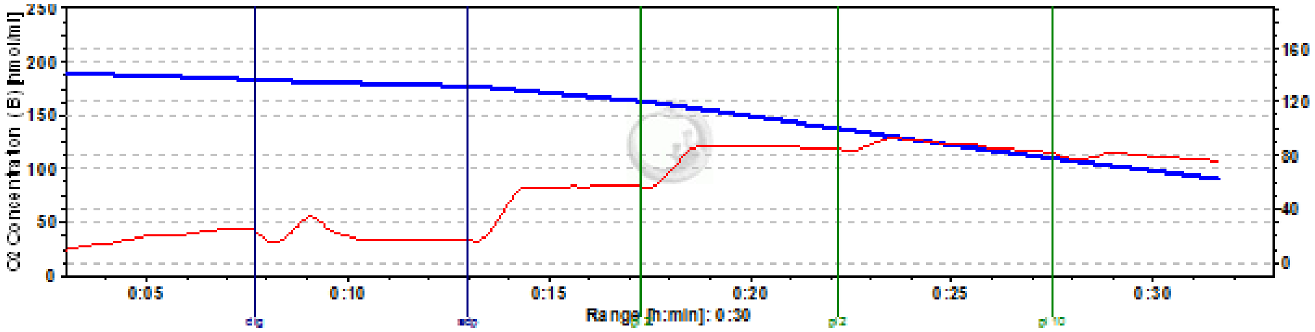

Figure S 1B. Typical traces of measurement of mitochondrial oxidative phosphorylation rate in C6 cell.

At the beginning respiration rate with 2×10^6 cells/chamber and in the presence of glutamate + malate (5 mM each) and succinate (12 mM) was recorded. Further additions: 10 µM of digitonin, 1 mM of ADP; 2 µM plumbagin, 2 µM plumbagin, 10 µM plumbagin . The upper trace (blue) indicates oxygen concentration in a respiratory chamber, the lower (red) one – oxygen consumption rate.
